# Visual Difficulty and Employment Status in the World

**DOI:** 10.1371/journal.pone.0088306

**Published:** 2014-02-07

**Authors:** Hanen Harrabi, Marie-Josee Aubin, Maria Victoria Zunzunegui, Slim Haddad, Ellen E. Freeman

**Affiliations:** 1 Centre de Recherche, Hôpital Maisonneuve-Rosemont, Montreal, Quebec, Canada; 2 Department of Ophthalmology, Université de Montréal, Montreal, Quebec, Canada; 3 Centre de Recherche de Centre Hospitalier de l’Université de Montréal, Montreal, Quebec, Canada; Zhongshan Ophthalmic Center, China

## Abstract

**Purpose:**

Using a world-wide, population-based dataset, we sought to examine the relationship between visual difficulty and employment status.

**Methods:**

The World Health Survey was conducted in 70 countries throughout the world in 2003 using a random, multi-stage, stratified, cluster sampling design. Far vision was assessed by asking about the level of difficulty in seeing and recognizing a person you know across the road (i.e. from a distance of about 20 meters). Responses included none, mild, moderate, severe, or extreme/unable. Participants were asked about their current job, and if they were not working, the reason why (unable to find job, ill health, homemaker, studies, unpaid work, other). The occupation in the last 12 months was obtained. Multinomial regression was used accounting for the complex survey design.

**Results:**

Of those who wanted to work, 79% of those with severe visual difficulty and 64% of those with extreme visual difficulty were actually working. People who had moderate, severe, or extreme visual difficulty had a higher odds of not working due to an inability to find a job and of not working due to ill health after adjusting for demographic and health factors (P<0.05).

**Conclusions:**

As the major causes of visual impairment in the world are uncorrected refractive error and cataract, countries are losing a great deal of labor productivity by failing to provide for the vision health needs of their citizens and failing to help them integrate into the workforce.

## Introduction

Unemployment not only causes a loss of income but is also recognized to have significant health impacts on adults and their children [1]–[5]. Advances in technology and low vision rehabilitation skills training have made it easier for blind and partially sighted people to carry out many employment activities. Unfortunately, studies indicate that unemployment rates in people with limited vision are high [6]–[8] and that job satisfaction is low [9]. However, limited population-based data on the employment status of people with visual difficulties are available. The World Health Survey, which provided population-based data from adults ages 18 and older from 70 countries of the world, allows us to examine employment in people with and without visual difficulty.

## Methods

### World Health Survey

#### Ethics statement

The project was conducted by the World Health Organization according to the principles expressed in the Declaration of Helsinki. Written informed consent was obtained from all participants. Ethics approval was obtained by the local institutional review committee in each of the 70 countries, and also from the Comité d’éthique of Hôpital Maisonneuve-Rosemont, where the analyses were conducted. Many papers have now been published using this global dataset [10]–[14].

#### Study population

The goal of the World Health Survey (WHS) was to collect population-based, nationally representative, cross-sectional data from 70 countries within 6 world regions. Data were collected from 276,647 adults ages 18 and older from 30 European countries, 18 African countries, 7 North and South American, 4 Eastern Mediterranean, 5 Southeast Asian, and 6 Western Pacific countries in 2002–2003. The institutions that carried out the surveys were selected by the World Health Organization (WHO) in each country. These institutions carried out the survey according to WHS procedures.

#### Sampling strategy

A multi-stage stratified random cluster sampling strategy was used to identify the participants to be contacted in each country. Strata were created based on 3 factors: region, socioeconomic status, and presence of a healthcare facility. Lists of households were obtained from population registries, voter lists, manual enumeration, or other methods. Households within the sampling units were randomly selected from these lists. Within each household, an adult 18 years or older was randomly selected using a Kish table to complete the survey. If the selected member of the household was in an institution, the survey team travelled to the institution. Non-response was carefully documented.

#### Survey administration

All surveys were administered by an interviewer in person in local languages. Questionnaires were translated into 68 local languages using standard techniques including forward and backward translation. Any discrepancies in the translations were resolved. A panel of experts then reviewed the translated document.

#### Vision data and its validity

Far vision was assessed by asking “In the last 30 days, how much difficulty did you have in seeing and recognizing a person you know across the road (i.e. from a distance of about 20 meters)?”. Possible responses included none, mild, moderate, severe, and extreme/unable. If the person wore glasses or contact lenses, the question was prefaced by asking the person to assess their difficulty while wearing their glasses or contact lenses as prescribed. We examined the validity of this WHS question in a sample of 139 patients recruited from an ophthalmology clinic in Montreal, Canada, as previously presented in Freeman et al [14]. The logarithm of the minimum angle of resolution (logMAR) visual acuity of these patients ranged from 0.0 (i.e. normal or 20/20) to 1.5 (i.e. blind or 20/632). Responses to the question were moderately correlated with logMAR visual acuity (Pearson’s r = 0.57, P<0.05). The mean logMAR scores of those who reported none, mild, moderate, severe, and extreme visual difficulty were 0.12, 0.30, 0.36, 0,52, and 0.60. Thus, a response of mild visual difficulty or worse may have closely corresponded to North American definitions of visual impairment (20/40 or 0.30 logMAR), while a response of severe visual difficulty may have corresponded to WHO definitions of visual impairment (20/60 or 0.48 logMAR).

#### Employment status

Participants were asked about their current job. If they were not working, reasons were ascertained such as: homemaker/caring for family, looked but cannot find job, doing unpaid work, studies/training, retired/too old to work, ill health, other. If they were working, the main occupation over the last 12 months was collected. We compared those who were working to those who were not working due to having “looked but been unable to find a job” or due to “ill health”. People who were not working due to the other reasons listed above were excluded because it was considered unlikely that the other reasons would be affected by vision. We examined “not working due to ill health” as an outcome because some people with poor vision might not have even tried to find a job because of their visual limitations, especially in countries with no laws protecting against employment discrimination and no low vision rehabilitation programs.

#### Additional data collection

Demographic information was collected on age, gender, and highest level of formal education completed. Participants were asked to rate their general health status on a scale from very good to very bad. They were asked to rate their level of difficulty with concentrating or remembering things and to rate their problems with feeling sad, low, or depressed.

### Data Compilation and Cleaning

The WHS datasets were downloaded for each country from the WHS website (http://www.who.int/healthinfo/survey/en/index.html) and appended together to create a single dataset. The data were checked and cleaned to eliminate implausible values, ineligible persons, and to exclude certain people without sufficient data. For example, 9,571 people were excluded who did not answer any questions in the individual questionnaire and 367 people were excluded who were listed as less than 18 years old. There were 32 people with ages between 100 and 120 years. Although some of these values may be imprecise due to the lack of birth records for older adults in many countries, we retained these values in the dataset because they are plausible values. The dataset after cleaning contained 276,647 people. Eleven countries (Austria, Belgium, Germany, Denmark, United Kingdom, Greece, Italy, Netherlands, Slovenia, Guatemala, Zambia) did not report survey design information (strata, primary sampling unit, and weights) and were therefore excluded giving 256,286 people from 59 countries.

### Data Analysis

Of 256,286 people from 59 countries, 219,048 people (85%) answered the questions on far vision and occupation. Comparisons were made between people who answered these questions and people who did not (Table 1). We then compared people who were working (n = 124,461) to those who were not due to a self- report of being unable to find a job (n = 11,075) or due to ill health (n = 3,418). We excluded people who were not working due to other reasons that we did not expect to be related to vision (e.g. homemaker, doing unpaid work, studies/training, retired/too old to work, other). Means, standard errors, and percentages were estimated.

**Table 1:** Descriptive characteristics of those included and excluded from analysis.

Variable	Categories	Included n = 219,048 (86%)	Excluded n = 37,238 (14%)	P-value
Age, mean (SE)		38.88 (0.10)	40.28 (0.45)	<0.001
Age Range, years		18–120	18–120	
Gender	Men	50.82	39.91	<0.001
	Women	49.18	60.09	
Education	> = Secondary School	44.43	54.51	<0.001
	Primary School	19.17	18.53	
	<Primary School	11.16	8.07	
	No Formal Education	25.25	18.89	
General Health Status	Very Good	22.03	17.15	<0.001
	Good	39.96	41.91	
	Moderate	28.67	31.88	
	Bad	7.83	7.89	
	Very Bad	1.51	1.17	
Cognitive Difficulties	None	62.94	65.83	<0.001
	Mild	19.81	17.07	
	Moderate	11.01	11.14	
	Severe	5.08	5.05	
	Extreme	1.17	0.91	
Depression	None	57.65	56.56	0.001
	Mild	22.19	20.87	
	Moderate	12.15	13.68	
	Severe	6.17	6.73	
	Extreme	1.85	2.16	

Differences in means and proportions were tested using Pearson’s chi square tests, linear regression, and simple multinomial regression. Multiple multinomial regression was performed to determine whether visual difficulty was associated with not working due to being unable to find a job or due to ill health while adjusting for other demographic and health factors. We adjusted for factors we thought would be associated with both vision and employment. These included demographic factors like age, gender, education, and health factors like general health status, cognitive difficulties, and depression. We chose these factors based on prior literature [15]–[18] and age and gender-stratified national employment statistics.

All analyses took into account the complex survey design by using the variables for sampling weight, strata, and primary sampling unit. The survey estimation (SVY) commands in STATA/IC software version 11.2 were used (StataCorp, College Station Texas, USA) with standard errors corrected using Taylor linearized variance estimation.

## Results

Those who did not answer the questions on occupation or far vision were compared to those who did (Table 1). Those who were excluded due to missing data were older, more likely to be women, had more education, were in slightly worse health, were slightly less likely to have cognitive difficulties, and were slightly more depressed (P<0.05). Almost half of the population (42.6%) was not working. The leading reasons for not working were: homemaker (50.0%), retired/too old (18.7%), looked but can’t find job (12.2%), studies/training (10.9%), ill health (3.8%), and other (4.4%).

Table 2 shows the factors that were associated with not working due to being unable to find a job or due to ill health. There was a clear, linear relationship between worsening visual difficulty and the prevalence of not working due to ill health. For example, 1.5% of those with no visual difficulty reported not working due to ill health while 13.0% and 26.1% of those with severe or extreme visual difficulty were not working due to ill health (P<0.05). The relationship between visual difficulty and having looked but been unable to find a job was slightly U-shaped without large differences between groups (P = 0.655).

**Table 2:** Employment status of WHS participants by selected characteristics.

Variable	Categories	Not Working Because Can’t Find Job* n = 11,075	P-Value†	Not Working Due toIll Health* n = 3,418	P-Value‡	Working n = 124,461
Far Visual Difficulty, %	None	8.15	0.655	1.48	<0.001	90.37
	Mild	6.31		3.90		89.78
	Moderate	7.75		7.69		84.56
	Severe	7.92		12.97		79.11
	Extreme	9.57		26.07		64.36
Age, mean (SE)		29.84 (0.25)	<0.001	50.51 (0.75)	<0.001	37.39 (0.10)
Gender, %	Men	6.77	<0.001	1.60	<0.001	91.63
	Women	10.37		4.20		85.43
Education, %	> = Secondary	9.00	<0.001	1.34	<0.001	89.66
	Primary	10.06		2.50		87.44
	<Primary	8.20		3.58		88.22
	No Formal Education	3.67		4.68		91.65
General Health Status, %	Very Good	8.81	0.610	0.19	<0.001	90.99
	Good	7.72		0.83		91.46
	Moderate	7.70		3.29		89.01
	Bad	7.45		14.90		77.65
	Very Bad	8.24		29.29		62.48
Cognitive Difficulties, %	None	7.78	<0.001	1.13	<0.001	91.09
	Mild	8.06		2.50		89.44
	Moderate	8.15		5.79		86.06
	Severe	10.35		13.22		76.44
	Extreme	8.85		26.82		64.33
Depression, %	None	7.14	<0.001	1.04	<0.001	91.82
	Mild	7.92		2.23		89.84
	Moderate	9.56		4.86		85.58
	Severe	12.10		10.89		77.02
	Extreme	16.25		17.10		66.65

*People not working due to other reasons were excluded.

† P-value from test comparing those not working because can’t find job to those working.

‡ P-value from test comparing those not working due to ill health to those working.

However, in a regression model adjusting for demographic and health factors, moderate (OR = 1.27, 95% CI 1.05, 1.55), severe (OR = 1.55, 95% CI 1.19, 2.01), and extreme (OR = 2.00, 95% CI 1.17, 3.40) visual difficulty were associated with having looked but been unable to find a job (Table 3). Other factors associated with having looking but been unable to find a job were younger age, female gender, and worse depressive symptoms (P<0.05). Those with no formal education were less likely to be unable to find a job compared to those who had completed secondary school (OR = 0.44, 95% CI 0.38, 0.52).

**Table 3:** Multiple multinomial regression results of relationship between visual difficulty and unemployment.

		Not Working Because Can’t Find Job*		Not Working Due to Ill Health*	
		OR	95% CI	OR	95% CI
Visual Difficulty	None	1.00		1.00	
	Mild	1.04	0.89, 1.21	1.11	0.90, 1.37
	Moderate	1.27	1.05, 1.55	1.48	1.06, 2.06
	Severe	1.55	1.19, 2.01	1.48	1.14, 1.92
	Extreme	2.00	1.17, 3.40	2.97	1.90, 4.64
Age	Per 1 year	0.94	0.94, 0.95	1.03	1.03, 1.04
Gender					
	Men	1.00		1.00	
	Women	1.59	1.45, 1.74	2.10	1.79, 2.46
Education	> = Secondary School	1.00		1.00	
	Primary School	1.13	0.99, 1.28	1.55	1.25, 1.93
	<Primary School	0.97	0.83, 1.13	1.40	1.09, 1.78
	No Formal Education	0.44	0.38, 0.52	1.43	1.15, 1.77
Cognitive Difficulties	None	1.00		1.00	1.00
	Mild	1.07	0.93, 1.22	1.12	0.89, 1.42
	Moderate	1.04	0.89, 1.22	1.41	1.11, 1.79
	Severe	1.29	0.99, 1.67	1.96	1.50, 2.56
	Extreme	1.06	0.62, 1.78	2.68	1.59, 4.52
General Health Status	Very Good	1.00		1.00	
	Good	0.91	0.81, 1.01	3.36	2.20, 5.13
	Moderate	0.97	0.85, 1.10	8.61	5.77, 12.83
	Bad	1.11	0.88, 1.41	24.04	15.63, 36.97
	Very Bad	1.31	0.78, 2.19	44.47	26.98, 73.31
Depression	None	1.00		1.00	
	Mild	1.20	1.07, 1.35	1.14	0.91, 1.42
	Moderate	1.55	1.32, 1.81	1.54	1.16, 2.02
	Severe	2.04	1.68, 2.48	2.37	1.83, 3.07
	Extreme	3.00	2.11, 4.26	2.83	1.83, 4.38

*People not working due to other reasons were excluded.

There were stronger relationships between visual difficulty and not working due to ill health as those with moderate (OR = 1.48, 95% CI 1.06, 2.06), severe (OR = 1.48, 95% CI1.14, 1.92) and extreme (OR = 2.97, 95% CI 1.90, 4.64) visual difficulty were more likely to not work due to ill health compared to those with no visual difficulty (Table 3). Other factors associated with not working due to ill health included older age, female gender, education less than secondary school, worse health status, worse depressive symptoms, and worse cognitive difficulties (P<0.05).

We compared the occupations of those with severe or extreme visual difficulty to those with moderate visual difficulty or less (P<0.05) (Table 4). There were two categories of jobs that were more common in those with severe or extreme visual difficulty: 43% of those with severe or extreme visual difficulty were working as an agricultural or fishery worker compared to 32% of those with moderate visual difficulty or less while 15% of people with severe or extreme visual difficulty were employed as an elementary worker (e.g. street food vendor, shoe cleaner) compared to 11% of those with moderate visual difficulty or less. People with severe or extreme visual difficulty were slightly underrepresented in all the other occupational categories.

**Table 4:** Type of occupation by visual difficulty status.

Occupation	None, Mild, or Moderate Visual Difficulty	Severe or Extreme Visual Difficulty
	Column %	Column %
Legislator, Senior Official, or Manager	1.62	1.11
Professional (engineer, doctor, teacher, clergy, etc.)	9.99	7.62
Technician or Associate Professional (inspector, finance dealer, etc.)	4.84	2.20
Clerk (secretary, cashier, etc.)	5.33	2.83
Service or sales worker (cook, travel guide, shop salesperson, etc.)	13.63	11.15
Agricultural or fishery worker (vegetable grower, livestock producer, etc.)	32.73	43.90
Craft or trades worker (carpenter, painter, jewelry worker, butcher, etc.)	11.24	9.94
Plant/machine operator or assembler (equipment assembler, sewing-machineoperator, driver, etc.)	8.18	4.84
Elementary worker (street food vendor, shoe cleaner, etc.)	11.28	15.41
Armed forces (government military)	1.17	1.00

## Discussion

Of people who wanted to work, we found that only 79% of people with severe visual difficulty and 64% of people with extreme visual difficulty were actually working (21% and 36% were not working, respectively). Furthermore, people with visual difficulty were more likely to have lower status jobs such as in the agricultural and fisheries professions or as an elementary worker. Because these are cross-sectional data, we do not know if people with greater visual difficulty were more likely to work in these professions because other jobs were not open to them or if these professions led to their greater visual difficulty. For example, people working in the agricultural or fisheries professions are exposed to high amounts of ultraviolet rays which are known to increase the risk of cortical cataract [19], [20]. They may also be at a higher risk of ocular irritation due to agrochemicals [21] [22] and ocular trauma [23].

Our results are in line with data from other studies. A convenience sample of 247 people with visual impairment in Victoria, Australia found that 73% of people who wanted to work were currently employed [8]. A population-based study using the British birth cohort found that worse habitual distance acuity was associated with not working due to “permanent sickness” with odds ratios that were between 1.5 and 4.6 depending on the level of visual impairment [6]. In the developing world, a cross-sectional study done in an onchocerciasis-endemic village in Guinea in 1987 found that 2%, 38%, and 79% of people who were sighted, impaired, or blind were not working, respectively [24].

The low rate of employment in people with limited vision is a concern for two reasons. First, although we do not know the cause of visual difficulty in this study, the leading causes of visual impairment in the world are due to conditions that are treatable such as uncorrected refractive error and cataract [25], [26]. If people are unemployed and therefore unable to financially provide for themselves and their families because of easily treatable conditions, this should be an absolute priority for governments to address. In fact, researchers have estimated that the global productivity loss due to uncorrected refractive error is between $269 and $428 billion and that it may actually result in economic gain to ensure appropriate eyeglasses for all working age adults [27]. The second reason that the low rate of employment in those with limited vision is a concern is that people with limited vision can still perform many employment activities with some additional accommodation and training. In fact, the “Americans with Disabilities Act” mandates that an employer with 15 or more employees provide reasonable accommodation to a person with a disability like poor vision [28]. Greater efforts across the world to help people with poor vision stay in the workforce are essential. In addition, employment status and job satisfaction should be included as secondary outcomes in clinical trials evaluating the efficacy of low vision rehabilitation programs in working age adults.

Several demographic factors were associated with the employment outcomes of interest. Younger age was associated with being unable to find a job while older age was associated with not working due to ill health. Youth unemployment has been called a public health emergency in Europe and efforts are needed to address this inequity [29]. Female gender was a risk factor for both being unable to find a job and not working due to ill health. Women are known to suffer from a higher incidence of disabling age-related chronic diseases that may lead them to prematurely drop out of the workforce [30]. In fact, in previous analyses using WHS data, we found that women had greater mobility difficulty than men, which could affect employment [31]. Also, inequalities in women’s access to education and gender discrimination may further contribute to our findings. Ironically, we found that people with no formal education were less likely to be unable to find work than those who had completed secondary school. Most people with no formal education work in physically demanding, low-skill jobs so although they are working, they often still live in poverty.

This paper is novel is its presentation of the employment status of people with and without vision problems using data from people from across the world. Additional strengths of this research include the population-based, random, cluster samples and the very large sample size of adults of different ages from many countries. Limitations include the lack of data on the cause of visual difficulty, the self-reported nature of the questions, and the cross-sectional nature of the data. Furthermore, some people did not answer questions on occupation or far vision leading to their exclusion from our analyses. However, only 14% of the sample was excluded and most of the differences between those who were included and those who were excluded were quite small as shown in Table 1.

From previous research, we know that the majority of visual impairment in the world is avoidable with the major causes of visual impairment being uncorrected refractive error and cataract, i.e. two treatable conditions [25], [26]. Furthermore, we previously reported that 38% of World Health Survey respondents had never had an eye exam including 61% in low income countries [32]. Therefore, countries may be losing a great deal of labor productivity by failing to provide for the vision health needs of their citizens and failing to help them integrate into the workforce.

## References

[1] Dupre ME, George LK, Liu G, Peterson ED (2012) The cumulative effect of unemployment on risks for acute myocardial infarction. Arch Intern Med 172: 1731–1737. 23401888 10.1001/2013.jamainternmed.447

[2] Roelfs DJ, Shor E, Davidson KW, Schwartz JE (2011) Losing life and livelihood: a systematic review and meta-analysis of unemployment and all-cause mortality. Soc Sci Med 72: 840–854. 21330027 10.1016/j.socscimed.2011.01.005 PMC3070776

[3] Stewart L, Liu Y, Rodriguez E (2012) Maternal unemployment and childhood overweight: is there a relationship? J Epidemiol Community Health 66: 641–646. 21422027 10.1136/jech.2010.110718

[4] Cohen F, Kemeny ME, Zegans LS, Johnson P, Kearney KA, et al (2007) Immune function declines with unemployment and recovers after stressor termination. Psychosom Med 69: 225–234. 17401058 10.1097/PSY.0b013e31803139a6

[5] Garcy AM, Vagero D (2012) The length of unemployment predicts mortality, differently in men and women, and by cause of death: a six year mortality follow-up of the Swedish 1992–1996 recession. Soc Sci Med 74: 1911–1920. 22465382 10.1016/j.socscimed.2012.01.034

[6] Rahi JS, Cumberland PM, Peckham CS (2009) Visual function in working-age adults: early life influences and associations with health and social outcomes. Ophthalmology 116: 1866–1871. 19560208 10.1016/j.ophtha.2009.03.007

[7] Szlyk JP, Fishman GA, Aslan RJ, Grover S (1998) Legal blindness and employment in patients with juvenile-onset macular dystrophies or achromatopsia. Retina 18: 360–367. 9730181 10.1097/00006982-199807000-00012

[8] McCarty CA, Burgess M, Keeffe JE (1999) Unemployment and under-employment in adults with vision impairment: The RVIB Employment Survey. Aust N Z J Ophthalmol 27: 190–193. 10484188 10.1046/j.1440-1606.1999.00194.x

[9] Mojon-Azzi SM, Sousa-Poza A, Mojon DS (2010) Impact of low vision on employment. Ophthalmologica 224: 381–388. 20606492 10.1159/000316688

[10] Hosseinpoor AR, Parker LA, Tursan d’Espaignet E, Chatterji S (2012) Socioeconomic inequality in smoking in low-income and middle-income countries: results from the World Health Survey. PLoS One 7: e42843. 22952617 10.1371/journal.pone.0042843 PMC3430638

[11] Loerbroks A, Herr RM, Subramanian S, Bosch JA (2012) The association of asthma and wheezing with major depressive episodes: an analysis of 245 727 women and men from 57 countries. Int J Epidemiol 41: 1436–1444. 22879363 10.1093/ije/dys123

[12] Moussavi S, Chatterji S, Verdes E, Tandon A, Patel V, et al (2007) Depression, chronic diseases, and decrements in health: results from the World Health Surveys. Lancet 370: 851–858. 17826170 10.1016/S0140-6736(07)61415-9

[13] Guthold R, Ono T, Strong KL, Chatterji S, Morabia A (2008) Worldwide variability in physical inactivity a 51-country survey. Am J Prev Med 34: 486–494. 18471584 10.1016/j.amepre.2008.02.013

[14] Freeman EE, Roy-Gagnon MH, Samson E, Haddad S, Aubin MJ, et al (2013) The global burden of visual difficulty in low, middle, and high income countries. PLoS One 8: e63315. 23675477 10.1371/journal.pone.0063315 PMC3651198

[15] Virtanen P, Janlert U, Hammarstrom A (2013) Health status and health behaviour as predictors of the occurrence of unemployment and prolonged unemployment. Public Health 127: 46–52. 23158056 10.1016/j.puhe.2012.10.016

[16] Ryskulova A, Turczyn K, Makuc DM, Cotch MF, Klein RJ, et al (2008) Self-reported age-related eye diseases and visual impairment in the United States: results of the 2002 national health interview survey. Am J Public Health 98: 454–461. 18235074 10.2105/AJPH.2006.098202 PMC2253577

[17] Popescu ML, Boisjoly H, Schmaltz H, Kergoat MJ, Rousseau J, et al (2012) Explaining the Relationship between Three Eye Diseases and Depressive Symptoms in Older Adults. Invest Ophthalmol Vis Sci 53: 2308–2313. 22427589 10.1167/iovs.11-9330

[18] Clemons TE, Rankin MW, McBee WL (2006) Cognitive impairment in the Age-Related Eye Disease Study: AREDS report no. 16. Arch Ophthalmol 124: 537–543. 16606880 10.1001/archopht.124.4.537 PMC1472655

[19] Cruickshanks KJ, Klein BE, Klein R (1992) Ultraviolet light exposure and lens opacities: the Beaver Dam Eye Study. Am J Public Health 82: 1658–1662. 1456342 10.2105/ajph.82.12.1658 PMC1694542

[20] West SK, Duncan DD, Munoz B, Rubin GS, Fried LP, et al (1998) Sunlight exposure and risk of lens opacities in a population-based study: the Salisbury Eye Evaluation project. JAMA 280: 714–718. 9728643 10.1001/jama.280.8.714

[21] Wesseling C, Hogstedt C, Fernandez P, Ahlbom A (2001) Time trends of occupational pesticide-related injuries in Costa Rica, 1982–1992. Int J Occup Environ Health 7: 1–6. 11210006 10.1179/107735201800339623

[22] Centers for Disease Control and Prevention (1995) Eye injuries to agricultural workers–Minnesota, 1992–1993. MMWR Morb Mortal Wkly Rep 44: 364–366. 7731453

[23] Alfaro DV 3rd, Jablon EP, Rodriguez Fontal M, Villalba SJ, Morris RE, et al (2005) Fishing-related ocular trauma. Am J Ophthalmol 139: 488–492. 15767058 10.1016/j.ajo.2004.10.011

[24] Evans TG (1995) Socioeconomic consequences of blinding onchocerciasis in west Africa. Bull World Health Organ 73: 495–506. 7554022 PMC2486790

[25] Resnikoff S, Pascolini D, Etya’ale D, Kocur I, Pararajasegaram R, et al (2004) Global data on visual impairment in the year 2002. Bull World Health Organ 82: 844–851. 15640920 PMC2623053

[26] Resnikoff S, Pascolini D, Mariotti SP, Pokharel GP (2008) Global magnitude of visual impairment caused by uncorrected refractive errors in 2004. Bull World Health Organ 86: 63–70. 18235892 10.2471/BLT.07.041210 PMC2647357

[27] Smith TS, Frick KD, Holden BA, Fricke TR, Naidoo KS (2009) Potential lost productivity resulting from the global burden of uncorrected refractive error. Bull World Health Organ 87: 431–437. 19565121 10.2471/BLT.08.055673 PMC2686211

[28] Karger H, Rose SR (2010) Revisiting the Americans with Disabilities Act after two decades. J Soc Work Disabil Rehabil 9: 73–86. 20730668 10.1080/1536710X.2010.493468

[29] Limb M (2011) Scale of youth unemployment is a public health emergency, Marmot says. BMJ 343: d7608. 22113573 10.1136/bmj.d7608

[30] Leveille SG, Penninx BW, Melzer D, Izmirlian G, Guralnik JM (2000) Sex differences in the prevalence of mobility disability in old age: the dynamics of incidence, recovery, and mortality. J Gerontol B Psychol Sci Soc Sci 55: S41–50. 10728129 10.1093/geronb/55.1.s41

[31] Mechakra-Tahiri SD, Freeman EE, Haddad S, Samson E, Zunzunegui MV (2012) The gender gap in mobility: A global cross-sectional study. BMC Public Health 12: 598. 22856611 10.1186/1471-2458-12-598 PMC3506530

[32] Vela C, Samson E, Zunzunegui M, Haddad S, Aubin M, et al.. (2012) Eye Care Utilization in Older Adults in Low, Middle, and High Income Countries. BMC Ophthalmology 12. 10.1186/1471-2415-12-5 PMC3378437 22471351

